# Evolution of a cross-feeding interaction following a key innovation in a long-term evolution experiment with *Escherichia coli*

**DOI:** 10.1099/mic.0.001390

**Published:** 2023-08-31

**Authors:** Caroline B. Turner, Zachary D. Blount, Daniel H. Mitchell, Richard E. Lenski

**Affiliations:** ^1^​ Ecology, Evolution and Behavior Program, Michigan State University, East Lansing, MI, USA; ^2^​ Department of Microbiology and Molecular Genetics, Michigan State University, East Lansing, MI, USA; ^3^​ Department of Microbiology and Molecular Genetics; and Ecology, Evolution and Behavior Program, Michigan State University, East Lansing, MI, USA; ^†^​ Present address: Department of Biology, Loyola University Chicago, Chicago, IL, USA; ^‡^​ Present address: Biological Sciences, University of New Hampshire, Durham, NH, USA

**Keywords:** adaptation, experimental evolution, cross-feeding interaction, evolutionary tinkering, citrate, C_4_-dicarboxylates

## Abstract

The evolution of a novel trait can profoundly change an organism’s effects on its environment, which can in turn affect the further evolution of that organism and any coexisting organisms. We examine these effects and feedbacks following the evolution of a novel function in the Long-Term Evolution Experiment (LTEE) with *Escherichia coli *. A characteristic feature of *E. coli* is its inability to grow aerobically on citrate (Cit^−^). Nonetheless, a Cit^+^ variant with this capacity evolved in one LTEE population after 31 000 generations. The Cit^+^ clade then coexisted stably with another clade that retained the ancestral Cit^−^ phenotype. This coexistence was shaped by the evolution of a cross-feeding relationship based on C_4_-dicarboxylic acids, particularly succinate, fumarate, and malate, that the Cit^+^ variants release into the medium. Both the Cit^−^ and Cit^+^ cells evolved to grow on these excreted resources. The evolution of aerobic growth on citrate thus led to a transition from an ecosystem based on a single limiting resource, glucose, to one with at least five resources that were either shared or partitioned between the two coexisting clades. Our findings show that evolutionary novelties can change environmental conditions in ways that facilitate diversity by altering ecosystem structure and the evolutionary trajectories of coexisting lineages.

## Data deposition

Data for this study are available at Data Dryad: https://doi.org/10.5061/dryad.sj3tx969q

## Introduction

Ecology and evolutionary biology are often viewed as separate fields. However, biologists since Darwin have recognized a continual interplay in which ecology sets the conditions under which evolution takes place, and evolution in turn alters ecological conditions [1–5]. Moreover, this feedback can occur even over short time scales [6–8].

The interplay between ecology and evolution can be particularly consequential when qualitatively new traits and functions evolve. These evolutionary innovations often allow organisms to exploit new resources and invade new niches, which can change environmental conditions, potentially driving diversification, speciation, and increased ecological complexity [9–12]. Perhaps the most dramatic example in the history of life on Earth was the evolution of oxygenic photosynthesis [13]. This new way of obtaining energy from sunlight was extraordinarily successful, but it produced oxygen as a toxic by-product, which over time oxygenated the atmosphere, disrupted geochemical cycles, and drove many organisms extinct [14–16]. Despite this upheaval, the resulting oxygenated world presented new ecological niches and opportunities that could be exploited by organisms that adapted to the changed conditions and evolved aerobic metabolism [14, 15]. Indeed, oxygenation set the stage for the later evolution of multicellularity and more complex forms of life [17].

Studying the eco-evolutionary effects of evolutionary innovations is usually difficult. Novel traits evolve only rarely, and the most dramatic examples arose in the distant past. Oxygenic photosynthesis evolved more than 3 billion years ago, so its consequences can only be inferred by studying fossil and geological records, from which the precise order of events is often difficult to discern [18]. A more tractable alternative is to examine the eco-evolutionary effects of less dramatic but nonetheless novel traits in present-day microbes. Novel traits more readily evolve in microbes owing to their large populations, short generations, and the availability of substantial variation via horizontal gene transfer [19–23]. Such innovations often confer the ability to grow on novel substrates, with the evolution of nylon-degradation being a prominent example [24].

Growth on novel substrates can have cascading ecological effects in microbial ecosystems owing in part to metabolic cross-feeding interactions, in which one strain produces molecules during its metabolism that another strain can then metabolize further [25]. Cross-feeding is common in nature, where it has important roles in microbial community assembly, function, diversification, and complexity [26–30]. In the human gut microbiome, for example, cross-feeding interactions can have far-reaching effects on health [31–35]. Cross-feeding interactions can evolve readily even in the absence of novel traits [36–40]. Indeed, theory predicts that cross-feeding is likely to evolve under certain conditions owing to physiological constraints that lead to evolutionary tradeoffs [41]. For instance, selection often favours bacterial variants that grow more quickly, but inefficiently, on a primary resource by partially degrading it and excreting the incompletely metabolized by-products [42]. These by-products then constitute a newly constructed niche that specialist variants can evolve to invade [25, 43, 44]. By allowing access to new resources, phenotypic innovations may provide even more extensive opportunities for the evolution of cross-feeding interactions that, in turn, can drive increased ecological complexity.

Novel traits can arise in tractable laboratory evolution experiments with microorganisms, providing ideal opportunities to examine the eco-evolutionary effects of those innovations. In these experiments, the fitness and other phenotypic properties of a living organism that possesses some innovation can be directly compared to those of its immediate progenitor that lacks the innovation. Moreover, these studies can be performed in the same environment in which the innovation evolved, under controlled conditions such that the innovation’s effects on that environment can be precisely determined, while molecular tools can be used to identify and manipulate relevant genetic changes. An opportunity to pursue this research programme occurred during the Long-Term Evolution Experiment (LTEE) with *Escherichia coli *.

Begun in 1988, the LTEE has followed twelve initially identical populations of *E. coli* for more than 75 000 generations, as they evolve in a carbon-limited medium, called DM25, in which glucose is the sole available substrate [45]. Despite these deliberately simple conditions, ecological complexity has repeatedly emerged during the experiment, including a well-characterized cross-feeding interaction that evolved in the population designated Ara−2 [46]. This interaction, based on acetate production during growth on glucose, has supported the stable coexistence of two lineages for tens of thousands of generations [47, 48]. Genomic data suggest that negative frequency-dependent interactions have allowed coexistence of multiple lineages in several other LTEE populations, probably also involving cross-feeding, for periods lasting from several thousand to tens of thousands of generations [49–51].

The DM25 medium also contains a high concentration of citrate, which is included as a chelating agent to help the bacteria acquire iron [52]. This citrate presents a potential second carbon and energy source. However, unlike many other bacteria, *E. coli* cannot grow aerobically on citrate (Cit^−^), and spontaneous mutants that can do so (Cit^+^) are extraordinarily rare [53–55]. Nonetheless, a weakly Cit^+^ variant evolved in one population, called Ara−3, around 31 000 generations [55], owing in part to a structural mutation that allowed expression of a previously unexpressed citrate transporter, CitT, during aerobic metabolism [55]. Replay experiments showed that the evolution of this new trait was historically contingent [55], with later work indicating that the new trait was contingent on an acetate cross-feeding interaction that had evolved early in the population’s history [56]. In particular, adaptation to acetate cross-feeding appears to have altered metabolism in a way that made access to citrate slightly beneficial, whereas it had been detrimental before [56]. Also, mutations with larger beneficial effects became increasingly rare in the LTEE as the populations continued to adapt over time [57], which enabled the weakly Cit^+^ variants to persist in the population [58].

By 33 000 generations, stronger Cit^+^ variants had evolved that were better able to exploit the citrate resource, driving a several-fold increase in the population’s size [55]. The successful Cit^+^ clade purged much of the diversity in the population, but a clade of bacteria that retained the ancestral Cit^−^ phenotype persisted [55, 59]. All of the Cit^−^ cells isolated after the Cit^+^ ecotype became numerically dominant come from the same clade, which had diverged from the clade that gave rise to the Cit^+^ lineage more than 10 000 generations before the innovation first arose [59].

How did the novel Cit^+^ trait change the population’s ecology? How did these changes affect the subsequent evolution of the Cit^+^ and Cit^−^ lineages? We address these questions here. We are particularly interested in examining any new cross-feeding interactions that might have evolved, because the mode of action of the CitT transporter suggests the potential for such interactions between Cit^+^ and Cit^−^ cells. CitT is a generalized di- and tricarboxylic acid antiporter that can import citrate, succinate, fumarate, and malate in exchange for the export of any of these same molecules [59–62]. Thus, Cit^+^ cells should export a molecule of succinate, fumarate, or malate for each net molecule of citrate imported, thereby generating a pool of C_4_-dicarboxylates in the environment that would provide an opportunity for cross-feeding. The LTEE ancestor does not grow appreciably on succinate, malate, or fumarate, but the potential to do so is present; evolved LTEE strains have been isolated that are able to grow on these substrates, including a Cit^+^ isolate from 50 000 generations that exhibits measurable growth on malate and succinate, though not on fumarate [63].

Here we show that the evolution of the ability to consume citrate has indeed altered the ecological conditions experienced by the bacteria, influencing the subsequent evolution of both the Cit^+^ and Cit^−^ lineages. In particular, we demonstrate that Cit^+^ cells release C_4_-dicarboxylates into the medium while they grow on citrate, modifying the environment and increasing its resource complexity. We also show that the coexisting Cit^−^ cells evolved to cross-feed on those new resources, but that doing so reduced their competitiveness for glucose and led to a reduction in fitness in the absence of Cit^+^ cells. However, the Cit^+^ lineage also evolved to use the new resources, and thus competed with the Cit^−^ lineage for both glucose and the C_4_-dicarboxylates that the Cit^+^ cells produce as they consume citrate.

## Methods

### Long-term evolution experiment with *E. coli*

The LTEE consists of 12 populations of *E. coli* B, which are serially propagated each day in 10 ml of Davis-Mingioli minimal medium that contains 25 mg l^−1^ glucose (DM25) [64]. Each population was started from one of two founder clones that differ by two neutral mutations, one of which enables cells to grow on the sugar arabinose. Cultures are grown at 37 °C with 120 r.p.m. orbital shaking under aerobic conditions. Growth in DM25 is carbon-limited; in the absence of growth on citrate, the ancestral bacteria reach a final population density of ~5 x 10^7^ cells ml^−1^. Cit^+^ cells, which remain carbon-limited (Fig. S1, available in the online version of this article), reach a higher final population density of ~2 x 10^8^ cells ml^−1^. Each population is diluted 1 : 100 into fresh medium daily, which allows populations to grow ~6.7 generations per day. Every 500 generations, samples of each population are frozen at −80 °C. The frozen cells remain viable and can be revived for later experimentation.

### Strains used and revival and pre-conditioning procedures

Besides the ancestral clone, REL606, all clones used in this study were isolated from the LTEE population, designated Ara–3, in which the Cit^+^ trait evolved. Unless otherwise specified, the Cit^−^ clones used in this study were from the Cit^−^ clade (called ‘clade 2’ in [59]) that persisted after the Cit^+^ ecotype became numerically dominant. All clones from the clade in which the Cit^+^ trait evolved (called ‘clade 3’ in [59]) used in this study that were isolated at 32 000 generations or later have a Cit^+^ phenotype; however, clones that were isolated from this clade at earlier time points have a Cit^−^ phenotype. Members of the Cit^−^ and Cit^+^ clades were identified by mutations unique to each lineage [59]. Table S1 lists all of the strains used in this study (Supplementary Material).

Prior to starting each experiment, we revived samples of frozen isolates by inoculating them into Lysogeny Broth (LB) and growing them overnight. We then pre-conditioned the cells to the environment of the LTEE by growing them for 2 days in DM25, with transfers into fresh medium every 24 h.

### Resource assays

Based on the mode of action of the CitT antiporter, we hypothesized that Cit^+^ cells release C_4_-dicarboxylates into the medium during growth on citrate. To test this hypothesis, we employed gas chromatography–mass spectrometry (GCMS) to measure the concentrations of succinate, fumarate, and malate in the medium over the course of the 24 h transfer cycle. We measured concentrations of these C_4_-dicarboxylates in a single culture of each of the following strains: REL606, the LTEE ancestor and founder of the Ara–3 population; a 40 000-generation clone from the Cit^−^ clade; and eight clones from the clade in which Cit^+^ evolved, which were isolated from samples taken every 2000 generations from 30 000 to 44 000 generations. Of the clones from the clade in which Cit^+^ evolved, the 30 000-generation clone has a Cit^−^ phenotype, the 32 000-generation clone exhibits weak growth on citrate, and the remaining six clones grow strongly on citrate. After reviving and pre-conditioning the clones as described above, we transferred the cultures to fresh DM25 medium. At 0, 2, 4, 6, 8, 12, and 24 h after transfer, 300 µl of each culture was collected using a sterile syringe, immediately filter-sterilized by passing the medium through a 0.25 µm filter, and frozen at −80 °C for later analysis.

We measured the succinate, fumarate, and malate concentrations in these samples using an Agilent 5975 GCMS system at the Michigan State University Research Technology Support Facility. Because DM25 medium contains a high concentration of phosphate that obscured all other molecules in full-scan mode, we could not conduct a broad chemical survey. We instead used selective ion measurement to isolate the spectral signatures specific to succinate, fumarate, and malate. Therefore, we cannot exclude the possibility that Cit^+^ cells release other molecules into the culture medium, especially since Cit^+^ cells have an elevated rate of cell death [65].

We also measured the concentration of citrate in the medium to compare the timing of citrate consumption to the timing of succinate, fumarate, and malate release. We measured citrate consumption by the 30 000-, 32 000-, 34 000-, and 40 000-generation clones from the lineage that produced the Cit^+^ trait with five-fold replication. We collected filtered samples of culture medium at 0, 1, 2, 3, 4, 6, 9, and 24 h after transfer, following the same protocol as above. We then measured the concentration of citrate in these samples using a Megazyme citric acid assay kit.

### Growth on C_4_-dicarboxylates

To assess changes in the ability of clones from the Cit^−^ and Cit^+^ clades to grow on succinate, fumarate, and malate, we analysed growth curves for Cit^−^ and Cit^+^ clones isolated every 1000 generations from 30 000 to 43 000 generations. This range includes clones from three periods of interest: (i) prior to the origin of the Cit^+^ phenotype (30000 to 31 000 generations); (ii) when Cit^+^ cells were present, but rare (32 000 to 33 000 generations); and (iii) after the Cit^+^ lineage rose to numerical dominance in the population (34 000 to 43 000 generations). Growth curves were obtained for three replicate cultures of each clone in the standard LTEE medium, except with a single alternative carbon source replacing glucose. In addition to the C_4_-dicarboxylates succinate, fumarate, and malate, we also measured the growth of the Cit^−^ clones on acetate, a common substrate for cross-feeding in *E. coli* [36, 37].

We used glucose as a carbon source during conditioning so that all cultures would have a similar initial density when transferred into the media used for measuring growth curves. For the Cit^−^ clones, growth curves were conducted in DM minimal medium supplemented with the relevant carbon source. For the Cit^+^ clones, growth curves were conducted in M9 minimal salts medium [66] supplemented with the relevant carbon source, in order to prevent the citrate in DM medium from being a confounding factor. The growth curves for the Cit^+^ and Cit^−^ clones are therefore not directly comparable. Instead, we can compare the growth curves within each group to understand the changes that evolved in each clade over time. We chose concentrations of the various carbon sources to give stationary-phase population densities similar to those in the glucose-limited DM25. These concentrations were as follows: succinate at 30.5 mg l^−1^, fumarate at 39.5 mg l^−1^, malate at 45.7 mg l^−1^, and sodium acetate at 34.4 mg l^−1^. The growth curves were generated in 96-well microplates by measuring optical density (OD) at 420 nm every 10 min for 24 h (or 48 h, where noted) using a VersaMax automated plate reader (Molecular Devices).

We analysed two characteristics of the growth curves: the final OD value at 24 h, and the time required to reach stationary phase. We determined the time to stationary phase by visually inspecting each log-transformed growth curve and identifying the time point when a population transitioned from growth to either a stable or declining OD. These visual assessments were done twice, in a blind fashion, and the results were highly consistent between the repetitions.

### Fitness assays

To determine how evolutionary changes in the Cit^−^ lineage affected the fitness of Cit^−^ cells in the presence and absence of Cit^+^ cells, we conducted a series of competition experiments between a reference 30 000-generation Cit^−^ clone and Cit^−^ clones sampled every 1000 generations from 30 000 to 40 000 generations. All of the Cit^−^ clones in these assays were from the same Cit^−^ clade (‘clade 2’ in [59]). These Cit^−^ clones competed against the reference clone in both the presence and absence of a mutant (described below) that was derived from a Cit^+^ clone sampled at 40 000 generations. Each competition was replicated three-fold. Competitions were performed over one standard 24 h cycle, and relative fitness values of the two Cit^−^ clones were calculated as the ratio of their realized population growth rates during the competition assay [45]. The Cit^−^ clones used in the competitions were the same as those used for the growth curves above, with the exception of the clone from generation 33 000, for which we inadvertently used CZB195 instead of CZB194. However, we confirmed in separate measurements that CZB195, like CZB194, is unable to grow on the C_4_-dicarboxylates.

The reference Cit^−^ competitor, called CBT1, is an arabinose-consuming (Ara^+^) mutant of a Cit^−^ clone isolated from the 30 000-generation population (Table S1). There is no arabinose in the LTEE medium; the ability to consume arabinose is selectively neutral under the conditions of the LTEE [45], but it allows discrimination between the colonies of different competitors based on their colour when plated on tetrazolium arabinose (TA) agar medium. Specifically, on TA plates, Ara^−^ clones form red colonies, while Ara^+^ clones form colonies that are white or pink [45, 67]. To isolate CBT1, we grew the 30 000-generation Cit^−^ clone in 10 ml of DM25 medium, then centrifuged the culture and spread the concentrated cells onto a minimal arabinose agar plate. After incubation, a single colony was chosen and purified by streaking twice on successive minimal arabinose plates. A colony from the second plate was grown in LB, and a sample of that culture was frozen. We then confirmed that CBT1 was selectively neutral compared to its parent clone by conducting a 7 day competition between the two clones under the same conditions as the LTEE.

To conduct competitions between pairs of Cit^−^ clones in the presence of Cit^+^ cells, we needed a way to prevent the Cit^+^ bacteria from growing on the TA plates. To this end, we used a bacteriophage λ-sensitive mutant, called CBT3, of a Cit^+^ clone isolated at 40 000 generations. Although the ancestor of the LTEE is sensitive to λ, resistant mutants were beneficial and went to fixation in population Ara–3 by 10 000 generations [68]. The resistant strains have reduced expression of LamB, a maltose transport protein that is also the adsorption site for λ [68, 69]. To isolate CBT3, we selected a maltose-consuming mutant following the procedure described above for isolating an Ara^+^ mutant, substituting minimal maltose for minimal arabinose agar plates. We confirmed that CBT3 was sensitive to phage λ by plating cells on TA plates with and without λ. The fitness of the Cit^+^ λ-sensitive mutant was similar to that of its λ-resistant parent in 1 day competition assays (relative fitness 0.98±0.06, mean±95 % confidence interval, *n*=10).

For all competitions between Cit^−^ clones that were performed in the presence of Cit^+^ cells, the cultures were plated together with an aliquot of phage λ lysate in order to prevent the growth of Cit^+^ colonies. However, a few Cit^+^ colonies grew on these plates, presumably due to incomplete coverage of λ on the plates or resistant mutants. To correct for this problem, we transferred a sample of all Ara^−^ colonies onto Christensen’s citrate agar, an indicator medium on which Cit^+^ bacteria cause a colour change from orange to bright pink [70]. Any colonies that showed a positive reaction on Christensen’s agar were excluded from the counts used to measure the relative fitness of the Cit^−^ competitors.

Based on the outcome of these experiments, we decided to compete Cit^−^ clones isolated at 33 000 and 34 000 generations directly against one another. We measured the relative fitness of the 33 000- and 34 000-generation clones with ten-fold replication in both the presence and absence of the Cit^+^ clone, following the same procedure as above. In half of the replicates, we competed the 33 000-generation clone against an Ara^+^ mutant of the 34 000-generation clone (named CBT2, isolated as described above); in the other half, we competed the 34 000-generation clone against an Ara^+^ mutant of the 33 000-generation clone (called CZB207).

### Genomic analysis

The genomes of seven clones from the Cit^−^ clade have been previously sequenced [59]. We examined these genome sequences to identify candidate mutations that might have affected the evolution of C_4_-dicarboxylate consumption. Three of the sequenced clones – ZDB357, ZDB200, and ZDB158 from generations 30 000 to 32 500 (Table S1) – could not grow on the C_4_-dicarboxylates. The remaining four clones – ZDB87, ZDB99, ZDB111, and REL10988 from generations 36 000 to 40 000 (Table S1) – exhibited strong growth on the C_4_-dicarboxylates. We used the breseq 0.23 software [71] to generate a list of mutations that were present in the clones that could grow on the C_4_-dicarboxylates, but absent in the clones that lacked that ability.

### Genetic manipulation

We constructed an isogenic derivative of the ancestral clone REL606 that carried the *dcuS* allele found in a 34000-generation Cit^−^ clone, called ZDB86. To do so, we used the pKOV plasmid and the methods in Link *et al*. [72]. We performed Sanger sequencing to confirm that the evolved *dcuS* allele was present in the resulting construct, called ZDB1052.

## Results

### Consumption of citrate and production of C_4_-dicarboxylates by Cit^+^ clones

**Fig. 1.**
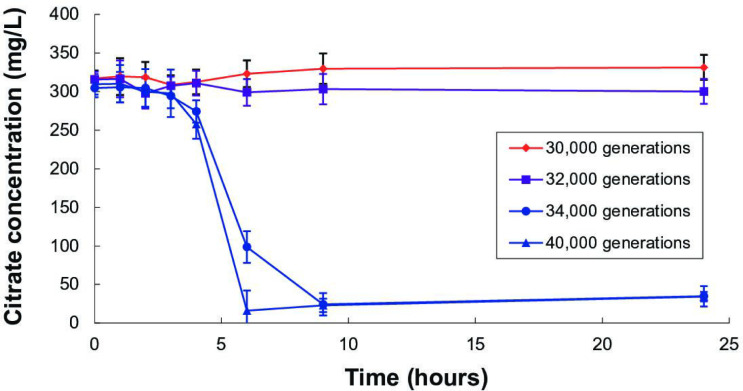
Citrate concentration in filtered medium sampled over the LTEE’s 24 h transfer cycle. Red points and lines show a Cit^−^ clone (30 000 generations), purple shows a weakly Cit^+^ clone (32 000 generations), and blue shows two strongly Cit^+^ clones (34 000 and 40 000 generations). Error bars are 95 % confidence intervals.

**Fig. 2.**
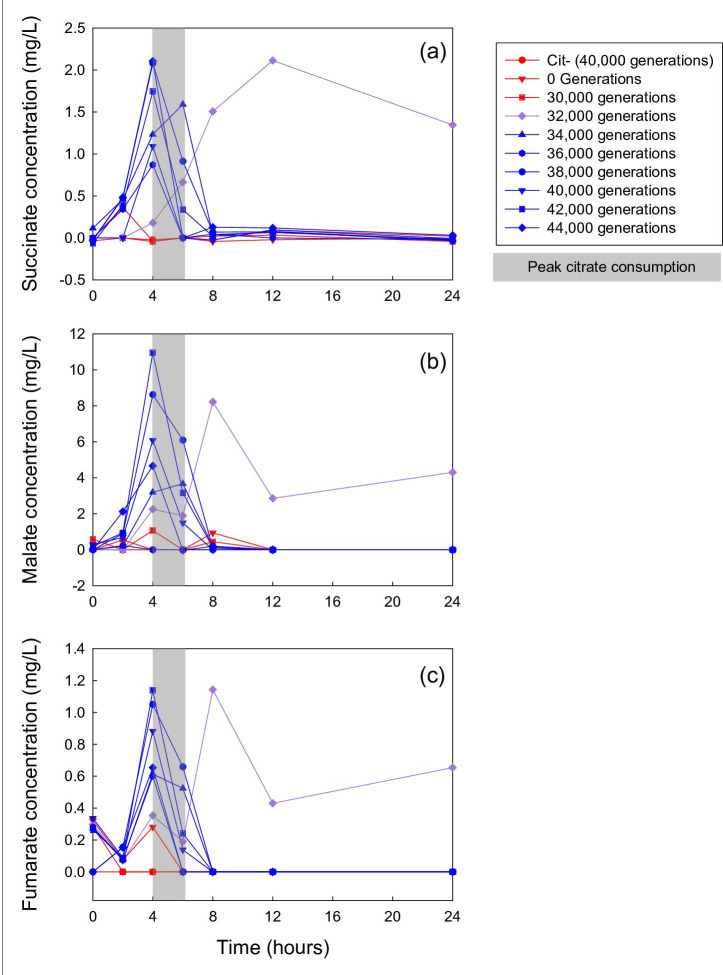
Concentrations of succinate (**a**), malate (**b**), and fumarate (**c**) in filtered medium sampled over the 24 h transfer cycle. Red points and lines show two Cit^−^ clones from the clade in which the Cit^+^ phenotype arose, purple shows a weakly Cit^+^ clone, and blue shows several strongly Cit^+^ clones. Grey boxes highlight the period of peak citrate consumption.

The Cit^+^ clones vary in the magnitude and timing of citrate consumption (Fig. 1). The earliest Cit^+^ clone (from generation 32 000) drew down the citrate only slightly, if at all, over the full 24 h transfer cycle. By contrast, both of the later Cit^+^ clones we tested (from generations 34 000 and 40 000) rapidly consumed the citrate between 4 and 6 h, which coincides with the period when the C_4_-dicarboxylates reached their highest levels (Fig. 2). By 9 h, both later clones had drawn down the citrate concentration to below the detection limit. These results are consistent with previous measurements of growth of these clones, where it was observed that the 32 000-generation Cit^+^ clone showed little increase in cell density compared to Cit^−^ clones, but the later Cit^+^ clones achieved densities at least five-fold higher [55].

Consistent with our expectations based on the mode of action of the CitT antiporter, the Cit^+^ clones excrete succinate, fumarate, and malate into the culture medium. The concentrations of these C_4_-dicarboxylates remained low throughout the 24 h transfer cycle in cultures of the ancestral strain (REL606), the 40 000-generation Cit^−^ clone, and a 30 000-generation clone from the clade that later gave rise to the Cit^+^ lineage (Fig. 2). By contrast, cultures of all of the Cit^+^ clones displayed elevated concentrations of both succinate and fumarate during at least part of the 24 h period (Fig. 2a, b). Malate concentrations were also noticeably elevated in cultures of all except one (from generation 36 000) of the Cit^+^ clones that we analysed (Fig. 2c).

Succinate levels peaked at 0.9 to 2.1 mg l^−1^ between 4 and 6 h after inoculation in cultures of Cit^+^ clones from 34 000 generations and later (Fig. 2a). Succinate levels then declined below the limit of detection by 8 h. By contrast, the succinate concentration in the culture of the 32 000-generation Cit^+^ clone, which grows only weakly on citrate, peaked at 2.1 mg l^−1^ at 12 h before declining to 1.3 mg l^−1^ at 24 h.

Fumarate and malate show similar patterns to succinate. Peak fumarate levels ranged from 0.6 to 1.1 mg l^−1^, while malate peaked between 3.2 and 11.0 mg l^−1^ (Fig. 2b, c). As with succinate, the 32 000-generation clone that grows only weakly on citrate released fumarate and malate, but it did not fully draw them down within 24 h. None of the C_4_-dicarboxylates showed any obvious trend, either upward or downward, in peak concentration over evolutionary time.

### Growth on C_4_-dicarboxylates

**Fig. 3.**
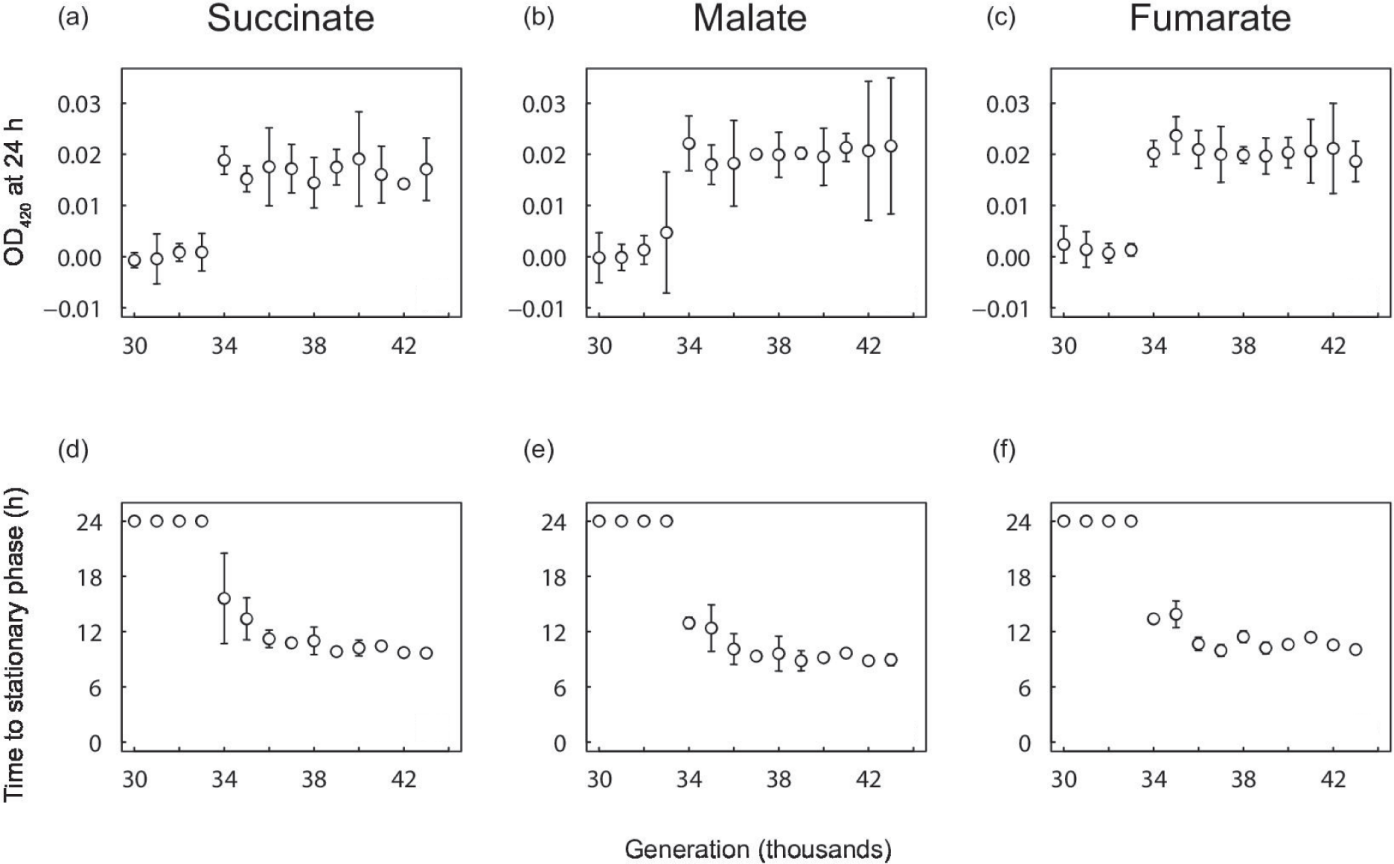
Final OD at 420 nm (**a-c**) and time to stationary phase (**d-f**) of clones isolated from the Cit^−^ clade at the various generations indicated, when grown in DM medium supplemented with 30.5 mg l^−1^ succinate, 39.5 mg l^−1^ fumarate, or 45.7 mg l^−1^ malate. If the bacteria did not reach a density consistent with depleting the available resource within 24 h, then the time to stationary phase is shown as 24 h. Error bars are 95 % confidence intervals.

The Cit^−^ clones that were sampled from 30 000 to 33 000 generations, and hence before Cit^+^ cells became numerically dominant in the population, did not exhibit a significant increase in OD after 24 h in glucose-free medium supplemented with any of the three C_4_-dicarboxylates (Figs 3 and S2). However, all of the Cit^−^ clones isolated from 34 000 generations and later showed improved growth on succinate, fumarate, and malate, with OD values substantially greater than zero after 24 h (Fig. 3a–c). These later clones also reached stationary phase before 24 h, with the time required to reach stationary phase declining between 34 000 and 43 000 generations (Fig. 3d–f).

**Fig. 4.**
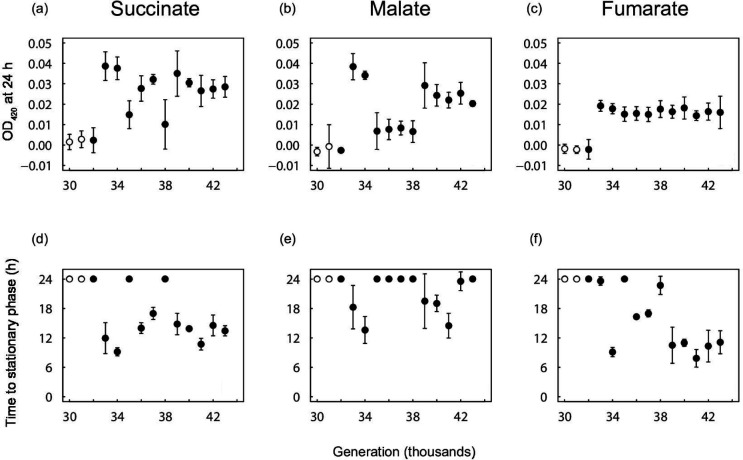
Final OD at 420 nm (**a-c**) and time to stationary phase (**d-f**) of clones isolated from the Cit^+^ clade at the various generations indicated, when grown in M9 medium supplemented with 30.5 mg l^−1^ succinate, 39.5 mg l^−1^ fumarate, or 45.7 mg l^−1^ malate. Open and filled circles show clones with Cit^−^ and Cit^+^ phenotypes, respectively. If the bacteria did not reach a density consistent with depleting the available resource within 24 h, then the time to stationary phase is shown as 24 h. Error bars are 95 % confidence intervals.

The clones isolated from the clade that later gave rise to the Cit^+^ trait showed negligible growth on C_4_-dicarboxylates through 32 000 generations (Figs 4 and S3). By contrast, Cit^+^ clones from 33 000 generations onward showed significantly improved growth on succinate, fumarate, and malate as sole carbon sources (Fig. 4a–c). The 32 000-generation clone has a weak Cit^+^ phenotype, but it did not grow on succinate, fumarate, or malate, indicating that the ability to use these carbon sources was not simply a pleiotropic effect of the mutations that allowed growth on citrate. In contrast to the clones from the Cit^−^ clade, we did not observe consistent improvement in growth on succinate by the Cit^+^ clones across the generations. For example, the Cit^+^ clones from generations 35 000 and 38 000 had lower final densities than other Cit^+^ clones, and they did not reach stationary phase by 24 h (Fig. 4d). The Cit^+^ clones showed similar variability on fumarate and malate (Fig. 4e, f), but the particular clones with slower growth or lower final densities differed across the three substrates.

**Fig. 5.**
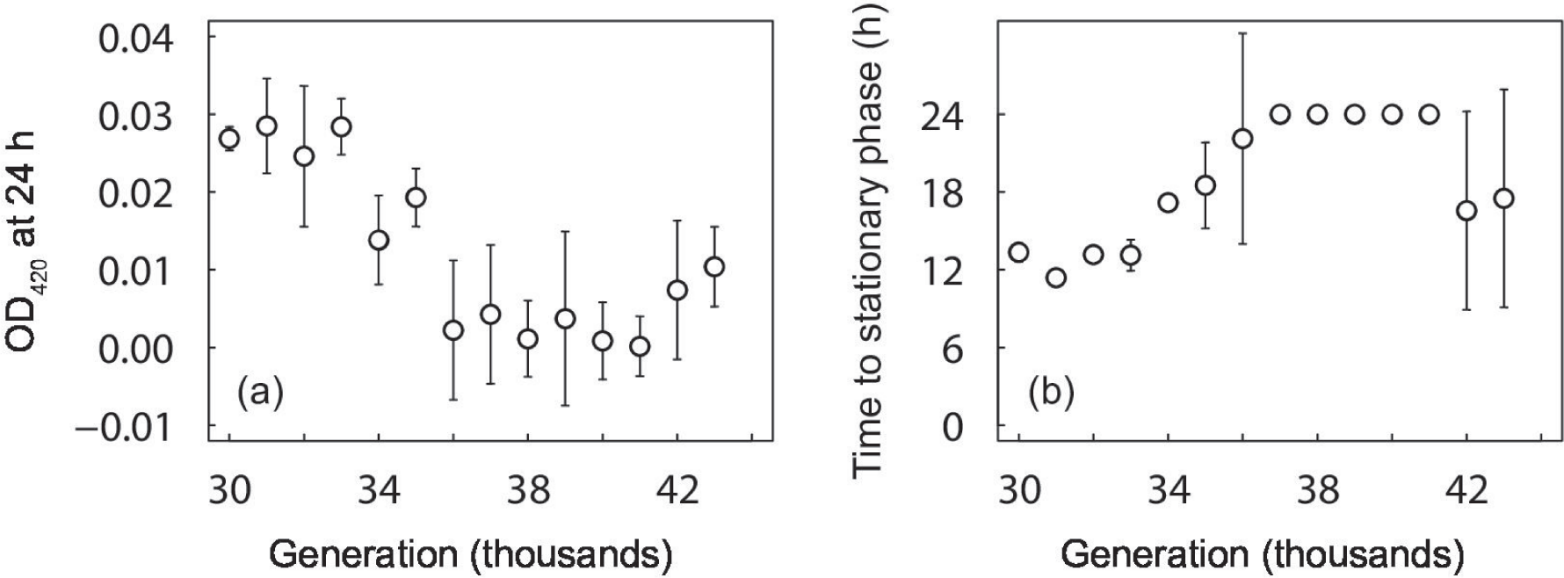
Final OD at 420 nm (**a**) and time to stationary phase (**b**) of clones from the Cit^−^ clade growing in DM medium supplemented with 30.5 mg l^−1^ acetate. If the bacteria did not reach a density consistent with depleting the available resource within 24 h, then the time to stationary phase is shown as 24 h. Error bars are 95 % confidence intervals.

In contrast to the improved growth on succinate, fumarate, and malate after the rise of the Cit^+^ lineage to numerical dominance, the clones from the Cit^−^ clade showed reduced growth on acetate, both in terms of final density (Fig. 5a) and time to stationary phase (Fig. 5b). Thus, there does not appear to have been selection for acetate cross-feeding by the Cit^−^ lineage. Moreover, the reduced growth on acetate indicates that the improved growth of Cit^−^ clones on C_4_-dicarboxylates is specific to those molecules, rather than being a general improvement in their growth or their ability to shift from growing on glucose to other carbon resources.

### Fitness of the Cit^−^ clones in the presence and absence of Cit^+^ cells

**Fig. 6.**
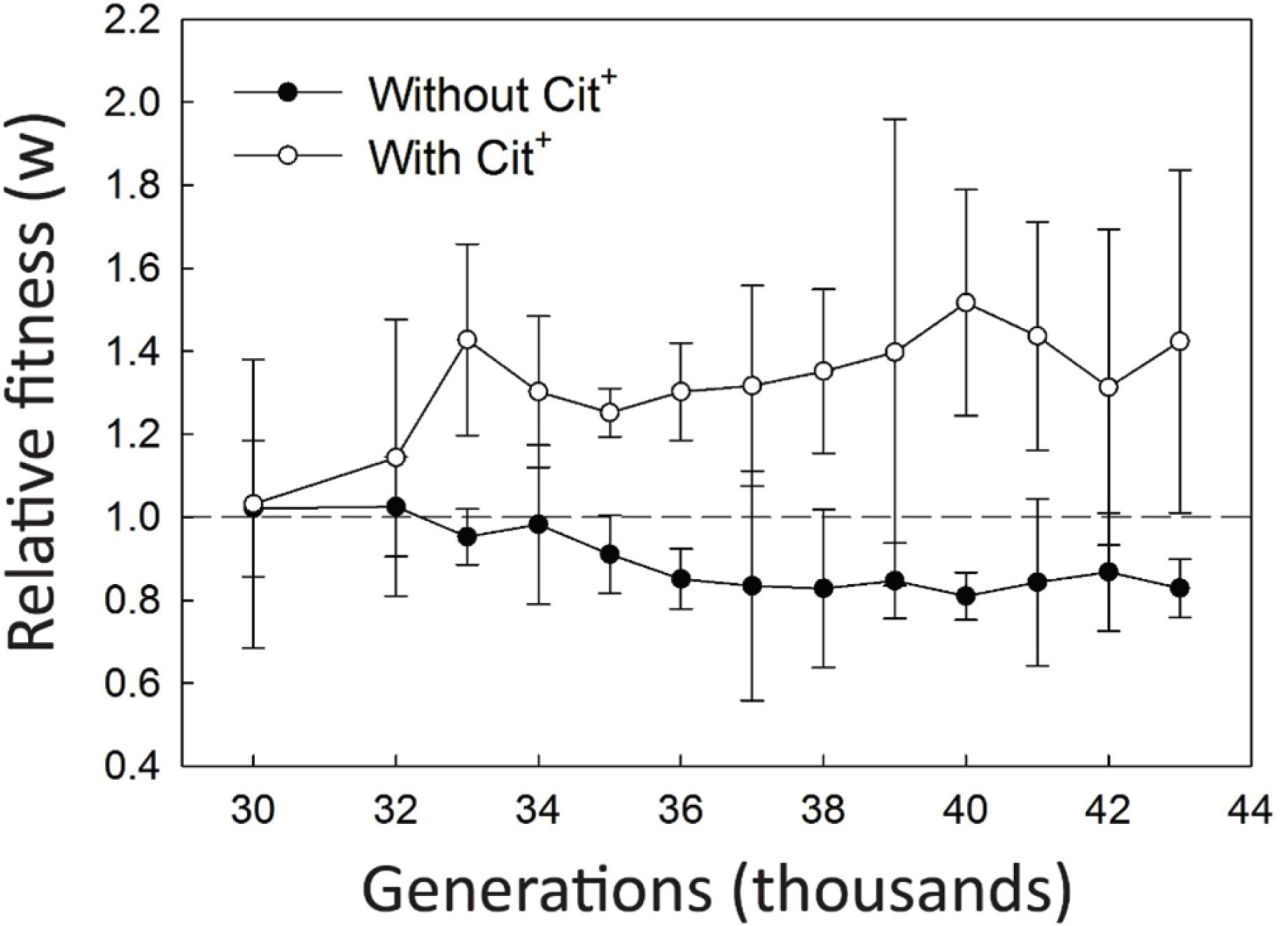
Fitness of clones sampled from the Cit^−^ clade at the generations indicated, relative to a 30000-generation clone from the Cit^−^ clade. Filled and open symbols indicate competitions between the Cit^−^ clones performed in the presence and absence, respectively, of a 40000-generation Cit^+^ clone. Error bars are 95 % confidence intervals.

If the Cit^−^ lineage evolved to feed on by-products excreted by the Cit^+^ lineage, then it should have higher fitness, relative to its Cit^−^ predecessors, in the presence of Cit^+^ cells than in their absence. We tested this prediction by competing clones from the Cit^−^ clade sampled at generations 30 000 and 32 000, and then at every 1000 generations from 33 000 to 43 000 generations, against a reference Cit^−^ clone from 30 000 generations, both in the presence and absence of a common Cit^+^ clone. The common Cit^+^ clone was excluded when counting the Cit^−^ competitors (see Methods), such that we can calculate the relative fitness of the Cit^−^ competitors even in the presence of the Cit^+^ clone. Consistent with our prediction, the fitness of the Cit^−^ clones trended higher in the presence of Cit^+^ clones in later generations, with mean fitness values relative to the 30 000-generation reference significantly greater than unity for most of the eleven time points we tested from generations 33 000 to 43 000 (Fig. 6, open symbols). Moreover, the fitness of the Cit^−^ clones actually declined over time in the absence of Cit^+^ clones (Fig. 6, filled symbols). These results not only support the cross-feeding hypothesis, they also imply that the adaptation of the Cit^−^ lineage to the cross-feeding niche imposed a tradeoff with respect to fitness on glucose, the sole resource available to their ancestor.

The overall trend in these data is largely consistent with our expectations. However, the increase in the fitness of the Cit^−^ clones, when assayed in the presence of Cit^+^ cells, seems to have begun by 33 000 generations (Fig. 6), whereas we did not see any evidence of their growth on C_4_-dicarboxylates until generation 34 000 and later (Fig. 3). If the differences in the fitness of the Cit^−^ cells in the presence and absence of Cit^+^ cells were caused by the evolution of growth on the C_4_-dicarboxylates, then the 34 000-generation C_4_-dicarboxylate-consuming Cit^−^ clone should be more fit than the 33 000-generation Cit^−^ clone in the presence of Cit^+^ cells. This discrepancy might suggest a more complicated scenario than envisioned in our hypothesis. Alternatively, it might be an artefact of the limited statistical resolution afforded by the three-fold replication for each combination of clone and treatment. To address this latter possibility, we directly competed the Cit^−^ clones from 33 000 and 34 000 generations against one another, and with ten-fold replication for each treatment, in order to better resolve how fitness changed over this critical period (see Methods and Table S1). Consistent with our prediction, when competed in the presence of Cit^+^ cells, the 34 000-generation C_4_-dicarboxylate-consuming Cit^−^ clone had a fitness of 1.14±0.04 (mean±95 % confidence interval) relative to the 33 000-generation clone that cannot use those substrates. Moreover, in the absence of the Cit^+^ cells, the later Cit^−^ clone had a fitness of only 0.94±0.02 relative to the earlier Cit^−^ clone, providing further support for the hypothesis that adaptation of the Cit^−^ cells to growth on C_4_-dicarboxylates imposed a tradeoff with respect to growth on glucose alone.

### Genome analysis of Cit^−^ clones

We compared the sequenced genomes of seven Cit^−^ clones that varied in their capacity for growth on C_4_-dicarboxylates, and we identified 19 mutations that might underlie that phenotype (Table S2). We then performed Sanger sequencing to determine the presence or absence of many of these mutations in six additional Cit^−^ clones: CZB193, CZB194, and CZB195, which did not grow on C_4_-dicarboxylates; and ZDB86, ZDB88, and ZDB92, which grew on those substrates. These additional data narrowed the list of candidates to nine mutations (Table S2). Of these candidates, the 5 bp deletion in the *dcuS* gene has the clearest relevance to the C4-dicarboxylate growth phenotype. The DcuS protein is a C_4_-dicarboxylate sensor that induces expression of the *dctA* gene, which in turn encodes DctA, a dicarboxylic-acid transporter [73]. The founding strain of the LTEE has a 5 bp insertion in *dcuS* [74], which introduces a frameshift that causes the expression of a shortened, non-functional version of DcuS and thereby precludes expression of DctA [75, 76].

### Effect of *dcuS* mutation on C4-dicarboxylate metabolism

We tested whether the deletion in *dcuS* was, in fact, a gain-of-function mutation that gave the Cit^−^ lineage the ability to grow on the C4-dicarboxylates that were excreted by the Cit^+^ lineage as it consumed citrate. To do so, we constructed ZDB1052, a derivative of the LTEE ancestor, REL606, that is isogenic except for the *dcuS* allele from a 34 000-generation Cit^−^ clone, called ZDB86. We then measured the growth trajectories over 48 h of REL606, ZDB86, and ZDB1052 on succinate, fumarate, and malate.

**Fig. 7.**
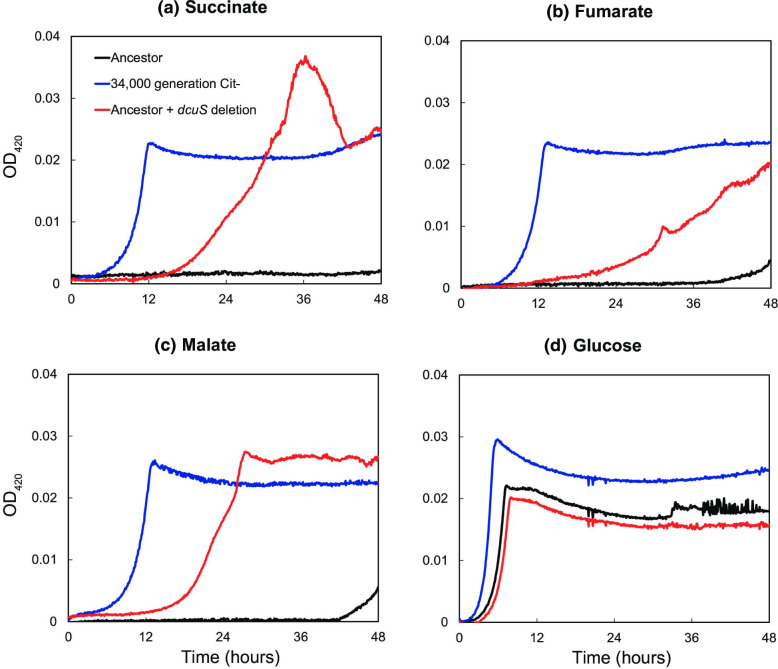
Growth curves showing OD at 420 nm of the LTEE ancestral strain (REL606, black), a 34000-generation Cit^−^ clone (ZDB86, blue), and a modified ancestral strain with the evolved *dcuS* allele (ZDB1052, red) grown in DM medium supplemented with (**a**) 30.5 mg l^−1^ succinate, (**b**) 39.5 mg l^−1^ fumarate, (**c**) 45.7 mg l^−1^ malate, or (**d**) 25 mg l^−1^ glucose.

ZDB1052 had improved growth on succinate, fumarate, and malate compared to REL606 (Fig. 7a–c). The densities achieved by ZDB1052 on these substrates after 48 h were comparable to those reached by ZDB86, the earliest Cit^−^ clone in our study to exhibit the C_4_-dicarboxylate growth phenotype and the source of the evolved *dcuS* allele. However, ZDB1052 grew more slowly on the C_4_-dicarboxylates than did ZDB86. Slower growth by ZDB1052 is not surprising, as it lacks many mutations that are beneficial for growth under the conditions of the LTEE, and which had fixed before the Cit^+^ trait evolved and C_4_-dicarboxylates appeared. Some of the additional mutations identified in Table S2 may also affect growth on C_4_-dicarboxylates; for example, the *arcB* gene encodes a protein that regulates enzymes involved in the citric acid cycle [77], of which C_4_-dicarboxylates are intermediates.

ZDB1052 grew more slowly on glucose, and it reached a slightly lower final density on that substrate, than did REL606, its counterpart with the ancestral *dcuS* allele (Fig. 7d). This result provides direct evidence that adaptation to growth on the C4-dicarboxylates imposes a tradeoff in terms of reduced growth on glucose, the sole carbon source available to the ancestral strain. ZDB86 does not show any such tradeoff compared to REL606 (Fig. 7d), but that is expected because it contains many additional mutations beneficial for growth on glucose.

REL606 did not grow appreciably on C_4_-dicarboxylates in the first 24 h, which corresponds to the interval between transfers in the LTEE. However, it did show slight growth between 36 and 48 h on two of these compounds (Fig. 7a–c). This pattern suggests that ancestral strain’s growth on C_4_-dicarboxylates, while not entirely absent, requires a long lag phase and is slow. Alternatively, this delayed growth could have resulted from a spontaneous mutant with the ability to grow on C_4_-dicarboxylates. It is perhaps relevant that the 5 bp insertion in *dcuS* in the ancestor creates a tandem array with three repeats of the same 5 bp sequence. The deletion of one copy of this repeat yields a two-copy array and a functional *dcuS* sequence. Such mutations in tandem arrays often occur at high rates [78]. In any case, we did not attempt to distinguish between these possibilities because the purpose of collecting these data was to test whether the evolved *dcuS* allele leads to improved growth on C_4_-dicarboxylates, which it clearly does.

## Discussion

The ability to grow aerobically on citrate has so far evolved in only one of the twelve LTEE populations [55], even after 75 000 generations [79] and billions of spontaneous mutations in each population [80]. By providing access to a previously unavailable resource, this evolutionary innovation caused dramatic ecological changes in the flask-based ecosystem where it arose. Owing to the abundant citrate in the medium, the total population size increased several-fold when the Cit^+^ cells became dominant in the population. However, the Cit^+^ lineage did not eliminate all of the non-citrate consumers from the population. Instead, a Cit^−^ ecotype persisted and coexisted for thousands of generations with the Cit^+^ ecotype in a frequency-dependent manner [55, 59]. In this study, we sought to understand the ecological dimensions of this prolonged coexistence, with a particular focus on any cross-feeding interactions that might have evolved between the two ecotypes.

Based on several lines of evidence, we conclude that the Cit^−^ ecotype evolved a cross-feeding relationship with the Cit^+^ ecotype, an interaction based on C_4_-dicarboxylates that Cit^+^ cells release into the medium as they grow on citrate. This interaction is consistent with the previously reported negative frequency-dependent relative fitness of the Cit^+^ and Cit^−^ ecotypes [55]. We also showed that the fitness of the Cit^−^ ecotype increased over time in the LTEE medium, but only in the presence of the Cit^+^ ecotype (Fig. 6). In the absence of the Cit^+^ ecotype, the Cit^−^ ecotype actually lost fitness in that medium (Fig. 6). This finding supports our hypothesis that the Cit^−^ lineage adapted to changes in the environment caused by the activity of Cit^+^ cells. In particular, we demonstrated that Cit^+^ cells excreted C_4_-dicarboxylates, including succinate, fumarate, and malate, into the culture medium during growth on citrate; by contrast, Cit^−^ cells, whether from the persistent Cit^−^ clade or from the clade in which the Cit^+^ trait later evolved, did not (Fig. 2). The earliest Cit^+^ clone that we tested showed only weak growth on citrate [59], but it, too, released C_4_-dicarboxylates into the medium. It did not, however, fully draw them back down during the course of the 24 h transfer cycle, whereas Cit^+^ clones from later generations both released C_4_-dicarboxylates and drew them down below detectable levels.

Although the observed concentrations of the endogenously produced succinate, fumarate, and malate were low relative to the exogenously added concentrations of glucose and citrate, the total amount of C_4_-dicarboxylates excreted and available to support growth would probably be much higher if they could be integrated over the entire growth cycle. In fact, the CitT transporter exports one molecule out of the cell for each molecule that it imports into the cell [62]. While some antiporter events might involve the exchange of one citrate molecule for another, the Cit^+^ strains (except in their earliest form) eventually consumed all of the citrate that was available each day (Fig. 1). It follows logically, therefore, that the total quantity of C_4_-dicarboxylate molecules released into the medium must equal the number of citrate molecules consumed, implying substantial opportunity for growth on C_4_-dicarboxylates and, consequently, strong selection to improve growth on those resources. C_4_-dicarboxylate concentrations were generally highest from about 4 to 6 h after inoculation, which coincided with the period when citrate was being drawn down from the medium (Fig. 1), corroborating the biochemical evidence [62] that the release of C_4_-dicarboxylates occurs simultaneously with citrate uptake.

In addition to showing that the Cit^+^ population released C_4_-dicarboxylates into the medium, and that the Cit^−^ population had become more fit in the presence (but not the absence) of the Cit^+^ population, we also tested whether the Cit^−^ lineage evolved to exploit those C_4_-dicarboxylates. Indeed, we found that C_4_-dicarboxylate consumption by the Cit^−^ lineage evolved in conjunction with the rise of the Cit^+^ population to numerical dominance between 33 000 and 33 500 generations. Thus, clones sampled from the Cit^−^ lineage through 33 000 generations showed no measurable growth on succinate, fumarate, or malate, whereas clones from 34 000 generations and later grew well on all three C_4_-dicarboxylates (Fig. 3). Moreover, the time required for populations to reach their maximum density, which depends on both the length of the lag phase and the subsequent growth rate, declined in later generations, indicating continued adaptation of the Cit^−^ population to growth on C_4_-dicarboxylates. This improvement was not a generalized improvement in fitness, because the growth of the Cit^−^ clones on both glucose and acetate declined over this period.

The Cit^−^ lineage eventually went extinct, but it persisted in the population for more than 10 000 generations, coexisting with the Cit^+^ lineage in a negative frequency-dependent manner [81]. Our results speak to two possible mechanisms for this coexistence. One possibility is that Cit^−^ cells, while unable to use the citrate resource, were able to outcompete the Cit^+^ cells for glucose. Indeed, a comparison of the growth curves of Cit^−^ and Cit^+^ cells showed that the Cit^−^ cells had a shorter lag phase when growing under the conditions of the LTEE, implying that the Cit^−^ cells had a period of exclusive access to glucose [55]. The fact that over evolutionary time the Cit^−^ strains became worse competitors in the glucose-limited medium argues against this being the sole explanation for the persistence of the Cit^−^ lineage. It is plausible, however, that the Cit^+^ lineage might also lose fitness on glucose as it gains fitness on citrate, in which case the Cit^−^ lineage might retain its advantage on glucose. The second possible mechanism for coexistence is that the Cit^−^ cells might cross-feed on one or more resources released by the Cit^+^ cells. Our results demonstrate that cross-feeding on C_4_-dicarboxylates indeed contributed to the coexistence of the Cit^+^ and Cit^−^ lineages. These two mechanisms are not mutually exclusive, and they could also act together with other mechanisms, such as Cit^+^ cell death [65], to enable coexistence.

The Cit^+^ lineage also evolved improved growth on C_4_-dicarboxylates, although its gains were more sporadic than the improvement in the Cit^−^ lineage (Fig. 4). The earliest clone able to grow on citrate, from generation 32 000, did not show detectable growth on C_4_-dicarboxylates, indicating that improved growth on these compounds was not simply a pleiotropic effect of the ability to consume citrate. The greater variability of growth on C_4_-dicarboxylates among clones from the Cit^+^ lineage suggests that selection to use these secondary resources was either weaker or less effective in this lineage than in the Cit^−^ lineage. Citrate is more energetically valuable than any of the C_4_-dicarboxylates, and so this variability among the Cit^+^ clones may reflect the fact that mutations that improve growth on citrate would be favoured even if they reduce growth on these by-products.

In both the Cit^+^ and Cit^−^ lineages, the primary mechanism underlying the improved growth on C_4_-dicarboxylates is increased expression of the DctA transporter, which is a proton motive force-driven symporter that *E. coli* requires for C_4_-dicarboxylate import during aerobic metabolism [82]. The LTEE ancestor does not express DctA, owing to a 5 bp frameshift mutation that disrupts the gene, *dcuS*, that encodes its positive regulator [74, 83]. We have shown that a 5 bp deletion in *dcuS* occurred in the Cit^−^ lineage, which restored the reading frame and enabled growth on C_4_-dicarbolyates (Fig. 7). By contrast, Quandt *et al*. [76] showed that a mutation in the promoter region of the *dctA* gene – one that caused constitutive, high-level expression of DctA – conferred the ability to grow on C_4_-dicarboxylates in the Cit^+^ lineage.

The consumption by Cit^−^ cells of the C_4_-dicarboxylates released by Cit^+^ cells as they consume citrate is an example of cross-feeding. Typically, cross-feeding occurs when one strain evolves to grow faster by partially degrading a primary resource, leaving a secondary resource for another strain to consume. In this canonical scenario, incomplete degradation of the primary resource is adaptive because it allows faster growth on the primary resource [41, 42]. However, the evolution of cross-feeding between the Cit^+^ and Cit^−^ lineages represents a somewhat different scenario. The release of C_4_-dicarboxylates by the Cit^+^ cells does not itself appear to be beneficial to them. Indeed, while the Cit^−^ lineage evolved to use the C_4_-dicarboxylates, so too did the Cit^+^ lineage. Thus, unlike the typical cross-feeding relationship, the two ecotypes have evolved increased, not decreased, overlap in their resource usage.

This situation seems to be a consequence of evolutionary ‘bricolage’ [84], whereby evolution tinkered with existing components to solve the problem of citrate acquisition. The Cit^+^ lineage evolved the novel ability to grow aerobically on citrate via multiple mutations including, in particular, a tandem duplication that caused aerobic expression of a previously unexpressed CitT transporter [59]. Most *E. coli* strains can express the CitT protein only during anaerobic fermentation of citrate, and even then only when there is a second substrate available to provide reducing power [60, 62]. Succinate is an end-product of citrate fermentation, so a transporter that exchanges citrate for succinate has a clear benefit. However, in the LTEE’s Cit^+^ lineage, the CitT transporter is expressed under carbon-limited, aerobic conditions, where succinate and other C_4_-dicarboxylates provide a useful source of energy and carbon.

Many other bacterial species use proton or sodium gradients to power citrate transport [61, 85], rather than the metabolite exchange mediated by the CitT antiporter. Moreover, in the only other reported case in which aerobic growth on citrate by *E. coli* arose by spontaneous mutation [54], the import of citrate appears to have been powered by the proton motive force [86], although the precise genetic basis is unknown in that case. If the Cit^+^ ecotype in the LTEE were based on a proton- or sodium-citrate transporter, then it would not export C_4_-dicarboxylates. A hypothetical Cit^+^ variant that employs a proton or sodium gradient would probably be favoured over the LTEE Cit^+^ ecotype that releases valuable molecules, which can be lost to competitors. But as a consequence of evolutionary tinkering, or bricolage, the Cit^+^ lineage first evolved to express the previously unexpressed CitT antiporter under aerobic conditions [59], and then to recover the exported C_4_-dicarboxylates using the previously unexpressed DctA transporter [76]. While this solution seems not to have been the most elegant or efficient, it nonetheless proved highly successful for the Cit^+^ clade. At the same time, it provided an opportunity for the Cit^−^ lineage to expand its niche as well, evolving from a glucose specialist to a generalist that also consumed some of the C_4_-dicarboxylates released by the Cit^+^ cells. This niche expansion also likely played an important role in the persistence of the Cit^−^ ecotype.

## Supplementary Data

Supplementary material 1
