# Supplementary material for: Evolution of a cross-feeding interaction following a key innovation in a long-term evolution experiment with Escherichia coli
Source: Microbiology (Reading). 2023 Aug 31;169(8):001390. doi: 10.1099/mic.0.001390 (PMC10482366; doi:10.1099/mic.0.001390)
Supplement: Supplementary material 1 [file mic-169-1390-s001.pdf]

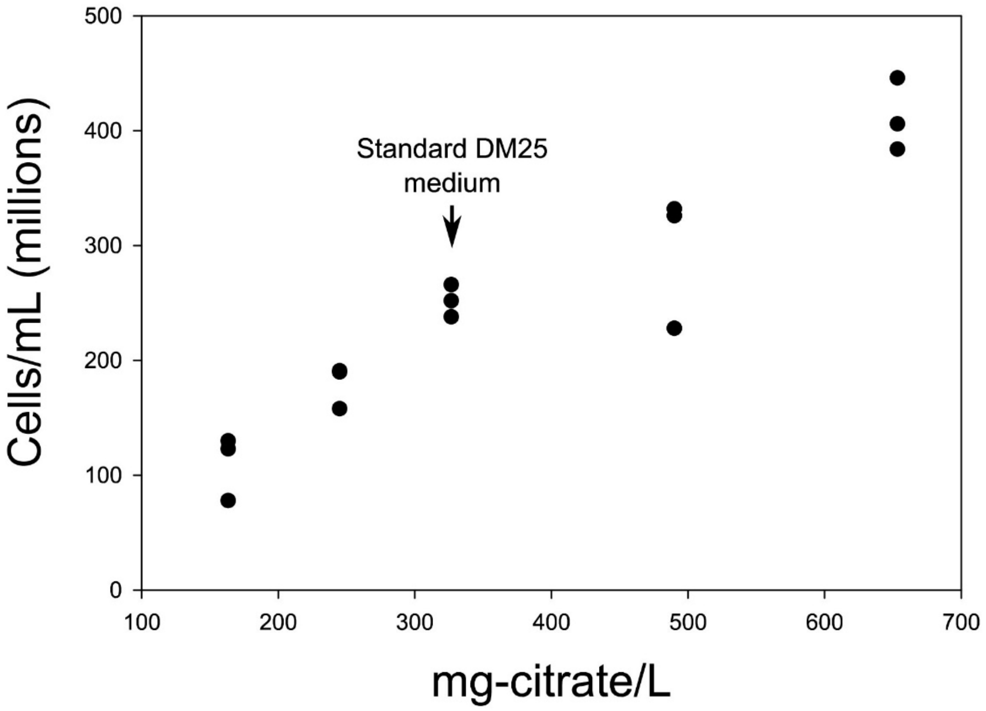

Figure S1. Stationary-phase density of a 40,000-generation Cit^{+} clone when grown in DM25 medium with varying citrate concentrations. Each point represents a different replicate culture. The continued increase in population density with increased carbon availability shows that the Cit^{+} population density is carbon limited. Cell densities were estimated from colony-forming units.

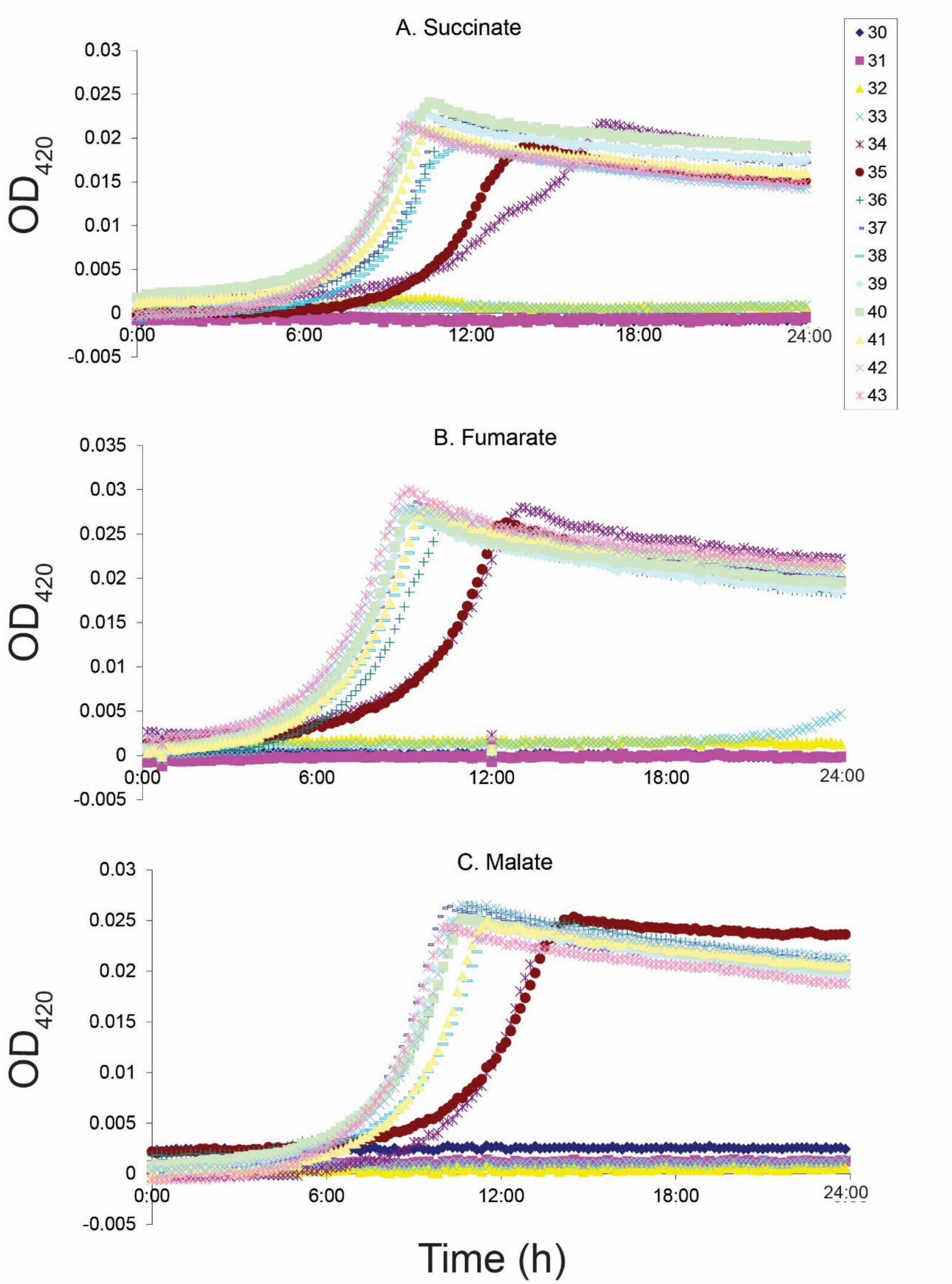

Figure S2. Growth curves of clones from the Cit^{-} lineage in DM medium supplemented with (A) succinate, (B) fumarate, or (C) malate. The legend shows the generation (in thousands) at which the clone was sampled. Each point is the average of three replicates.

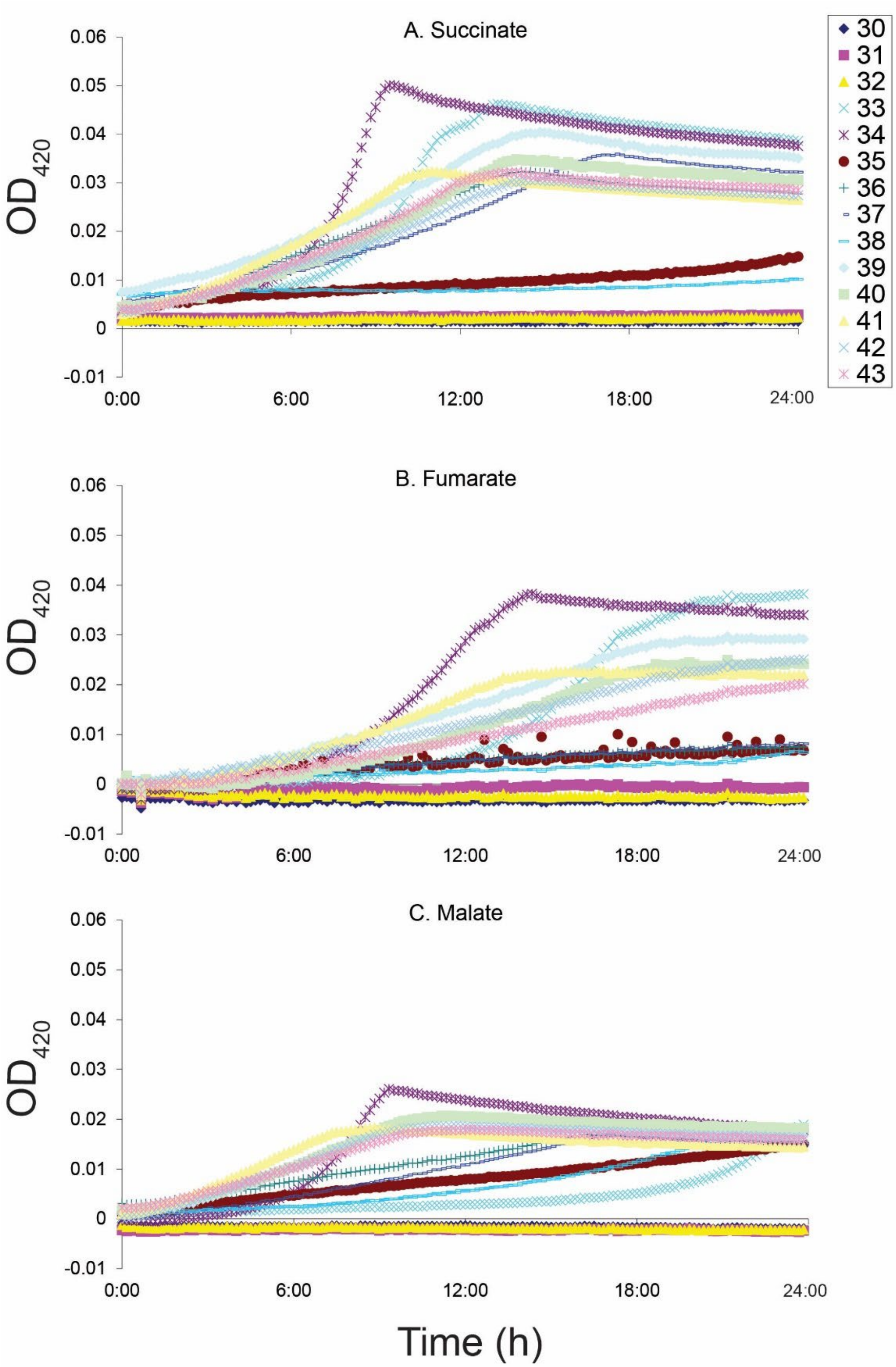

Figure S3. Growth curves of clones from the Cit$^+$ lineage in M9 medium supplemented with (A) succinate, (B) fumarate, or (C) malate. The legend shows the generation (in thousands) at which the clone was sampled. Each point is the average of three replicates.

Generation	ID Number	Description
0	REL606	Founder of Ara–3 population
30,000	ZDB357	Cit$^-$ clone
30,000	CBT1	Ara$^+$ mutant of ZDB357
31,000	ZDB374	Cit$^-$ clone
31,000	ZDB200	Cit$^-$ clone (sequencing only)
32,000	ZDB188	Cit$^-$ clone
32,500	ZDB158	Cit$^-$ clone (sequencing only)
33,000	CZB193	Cit$^-$ clone (sequencing only)
33,000	CZB194	Cit$^-$ clone (growth curves)
33,000	CZB195	Cit$^-$ clone (fitness measurements)
33,000	CZB207	Ara$^+$ mutant of CZB195
34,000	ZDB86	Cit$^-$ clone
34,000	CBT2	Ara$^+$ mutant of ZDB86
34,000	ZDB87	Cit$^-$ clone (sequencing only)
34,000	ZDB88	Cit$^-$ clone (sequencing only)
35,000	ZDB92	Cit$^-$ clone
36,000	ZDB99	Cit$^-$ clone
37,000	ZDB104	Cit$^-$ clone
38,000	ZDB111	Cit$^-$ clone
39,000	ZDB576	Cit$^-$ clone
40,000	REL10988	Cit$^-$ clone
41,000	ZDB597	Cit$^-$ clone
42,000	ZDB600	Cit$^-$ clone
43,000	ZDB606	Cit$^-$ clone
30,000	ZDB17	Cit$^-$ clone from Cit$^+$ clade
31,000	ZDB23	Cit$^-$ clone from Cit$^+$ clade
32,000	ZDB172	Cit$^+$ clone
33,000	CZB154	Cit$^+$ clone
34,000	ZDB83	Cit$^+$ clone
35,000	ZDB89	Cit$^+$ clone
36,000	ZDB96	Cit$^+$ clone
37,000	ZDB101	Cit$^+$ clone
38,000	ZDB107	Cit$^+$ clone
39,000	ZDB575	Cit$^+$ clone
40,000	REL10979	Cit$^+$ clone
40,000	CBT3	λ-sensitive mutant of REL10979
41,000	CBT4	Cit$^+$ clone
42,000	CBT7	Cit$^+$ clone
43,000	CBT10	Cit$^+$ clone
44,000	CBT13	Cit$^+$ clone

Table S1. List of the bacterial strains used in this study. Unless otherwise noted, all of the Cit$^-$ clones from generations 30,000 to 43,000 belong to the Cit$^-$ clade that persisted after the Cit$^+$ lineage became numerically dominant (Blount et al. 2012).

Position	Mutation	Annotation	Gene	Description
1,140,245	A→T	N38Y (AAT→TAT)	*rimJ* →	ribosomal-protein-S5-alanine N-acetyltransferase
1,761,565	G→A	E441K (GAG→AAG)	*ydiD* →	hypothetical protein
1,782,849	G→A	A167V (GCC→GTC)	*ydiY* ←	hypothetical protein
**2,191,174**	**IS*150* (+) +3 bp**	**coding (411-413/999 nt)**	***mglB* ←**	**methyl-galactoside transporter subunit**
2,507,912	A→C	T452P (ACC→CCC)	*narQ* →	sensory histidine kinase in two-component regulatory system with NarP (NarL)
**2,784,696**	**Δ1,396 bp**		***[ygcR]–[ygcS]***	***[ygcR], [ygcS]***
**2,899,574**	**IS*150* (+) +3 bp**	**coding (419-421/1626 nt)**	***yqeB* ←**	**conserved protein with NAD(P)-binding Rossman fold**
2,918,435	A→C	N439H (AAC→CAC)	*ygfU* →	predicted transporter
**2,983,794**	**C→T**	**D259D (GAC→GAT)**	***yggW* →**	**coproporphyrinogen III oxidase**
3,046,412	G→A	intergenic (-409/+77)	*yghJ* ← / ← *yghK*	predicted inner membrane lipoprotein/glycolate transporter
3,141,566	C→T	L242L (CTC→CTT)	*ygjE* →	predicted tartrate:succinate antiporter
**3,288,053**	**C→G**	**V70L (GTC→CTC)**	***arcB* ←**	**hybrid sensory histidine kinase in two-component regulatory system with ArcA**
**3,407,922**	**C→A**	**G197G (GGG→GGT)**	***kefB* ←**	**glutathione-regulated potassium-efflux system protein**
**3,570,410**	**A→C**	**intergenic (-1/-390)**	***yhiO* ← / → *uspA***	**universal stress protein UspB / universal stress global response regulator**
3,798,623	IS*1* (-) +9 bp	coding (444-452 / 924 nt)	*yicL* →	predicted inner membrane protein
4,164,385	G→A	R1177H (CGC → CAC)	*rpoB* →	DNA-directed RNA polymerase subunit beta
**4,313,510**	**C→T**	**A265A (GCG→GCA)**	***yjdB* ←**	**predicted metal dependent hydrolase**
**4,329,504**	**Δ5 bp**	**pseudogene (875-879/912 nt)**	***dcuS* ←**	**sensor of fumarate two-component regulatory system; b4125_2**
4,414,736	A→G	intergenic (+182 / -127)	*fklB* → / → *cycA*	FKBP-type peptidyl-prolyl cis-trans isomerase (rotamase) / D-alanine/D-serine/glycine transporter

Table S2. List of the mutations present in all four sequenced C_4-dicarboxylate-consuming Cit^{-} clones and absent from all three Cit^{-} clones that cannot grow on the C_4-dicarboxylates. The mutations in bold are the reduced set of candidates for conferring growth on C_4-dicarboxylates after performing Sanger sequencing of the relevant genes in additional clones. Arrows in the fourth column indicate the direction of transcription of the mutated gene.
